# Functional capacity and its associated factors in the elderly with low back pain

**DOI:** 10.1590/1413-78522014220600890

**Published:** 2014

**Authors:** Roger Palma, Marta Helena Souza de Conti, Natasha Mendonça Quintino, Marcia Aparecida Nuevo Gatti, Sandra Fiorelli Almeida Penteado Simeão, Alberto de Vitta

**Affiliations:** 1. Universidade Paulista, Bauru, SP, Brazil, Universidade Paulista (UNIP), Bauru, SP, Brazil; 2. Universidade do Sagrado Coração, Bauru, SP, Brazil, Universidade do Sagrado Coração (USC), Bauru, SP, Brazil

**Keywords:** Low back pain, Family Health, Activities of daily living, Personal autonomy, Aged, Risk factors

## Abstract

**OBJECTIVE:**

To assess the level of functional capacity in subjects aged 60 years and older, who have lower back pain, and its association with demographic, socioeconomic, work-related, lifestyle-related and disease mentioned variables.

**METHODS:**

A cross-sectional study was conducted with 246 elderly registered at the Family Health Strategy of Vila São Paulo, Bauru,SP, Brazil, who reported lower back pain and were sampled by a two-stage cluster technique. The subjects were interviewed at home by using a multidimensional instrument (demographic; socioeconomic aspects; life style; work characterization; disease mentioned), and also the IPAQ, the Nordic and the Roland Morris questionnaires. A bivariate and multivariate descriptive logistic regression analysis was carried out.

**RESULTS:**

The prevalence of lower back pain in men was of 25.1% and in women it was of 35.1%. The mean score in the functional capacity assessment was 10.46 ± 5.62. A fraction of 67.5% of the elderly demonstrated an inappropriate functional capacity. The age group from 70 to 80 years old, the subjects reporting three or more diseases and the sedentary group presented an independent association with inappropriate functional capacity.

**CONCLUSION:**

The older, sedentary subjects and who reported more than three diseases presented low functional capacity. **Level of Evidence III, Cross Sectioning.**

## INTRODUCTION

Low back pain is one of the most common musculoskeletal disorders in the world, affecting approximately 70% to 85% of the population at some time in life,^1^ and can cause large decrease of the construct "functional capacity" that indicates the maximum possible functionality that a person can achieve in a given time, i.e., it interferes with the autonomy and quality of life. Thus, the functional capacity emerges as an ideal value for the elderly in order to live independently and autonomously, being able to perform physical and mental activities necessary for maintaining his basic activities such as value: bathing, dressing, performing personal hygiene, transfer and move himself, getting up, eating, keeping continence, preparing meals, having financial control, etc.^2^

For the assessment of functional capacity of individuals with low back pain, various instruments are proposed in literature, such as the questionnaires "Roland Morris", "Oswestry Low Back Pain", "Disability Questionnaire", "Waddell Disability Index" and "Sickness Impact Profile". Among these, the Roland Morris questionnaire has been widely used in research and clinical practice since it has been translated, adapted and validated for the Brazilian population.^3^

The Family Health Strategy (FHS), implemented in Brazil in 1994, aims at the reorganization of primary care in the country, according to the precepts of the Unified Health System (SUS). The professionals involved in primary care should consider the National Policy and the Elderly Statute, which ensures healthy aging, through actions such as population surveys and intervention programs recommended by the Ministry of Health.^4^

Assessments such as functional capacity enable to provide information on the profile of the elderly constituting simple and useful tools in identifying the limitations and loss of autonomy of individuals. Through the functional capacity evaluation strategies to promote health of the elderly aiming at delaying or preventing disabilities can be defined.

Considering the theoretical framework, this study aimed to verify the level of functional ability in individuals aged 60 years and older with low back pain and its association with sociodemographic variables, work-related, lifestyle and referred diseases.

## MATERIALS AND METHODS

This is a cross-sectional study of a population of individuals aged 60 years and older of the areas covered by the FHS Vila São Paulo, in the city of Bauru, SP, Brazil.

The target population was limited to elderly above 60 years old living in areas circumscribed to the Family Health Strategy in the Vila São Paulo region, Northern region of Bauru, which has two FHS teams with eight community workers. This region consists of four districts: Vila São Paulo, Jardim Ivone, and Pousada da Esperança I and II, with an estimated population of 12,600 inhabitants.

The number of seniors enrolled in the FHS program was of 643 subjects. One sample per cluster was used in two-stages, the first stage comprised the two family health units distributed in Vila Sao Paulo - basic selection units - where the stratified sampling proportional to the amount of elderly enrolled for coverage area of ​​each community worker was performed. In the second stage, the elderly, considered the sample unit, were chosen randomly from the register of families seen by the health agent. Thus, the sample size was calculated considering the elderly population (643 individuals), the prevalence of 24%,^5^^,^ 6 a sampling error of 3% and a confidence level of 95%, estimating a total of 360 seniors in the FHS of the referred region, including 160 individuals from area 401 (neighborhoods Pousada da Esperança I and II) and 200 individuals from the area 701 (Vila São Paulo and Jardim Ivone). Among the 360 elderly, 246 reported low back pain, constituting, then, the subject of the present study.

The subjects were informed of all stages of the study, the voluntary nature of participation, the possibility to leave the survey at any time and the right to confidentiality of individual data. Those who agreed to participate in the research signed a Free and Informed Consent.

Data collection was carried out between December 2011 and March 2012. The research was approved by the Research Ethics Committee of Universidade do Sagrado Coração - Proc. nº 201/11.

The researchers visited the households covered by the FHS accompanied by the community workers of each sector. Interviews with randomly selected subjects were performed and were excluded elderly unable to answer the questionnaires, such as mentally disabled and individuals who had suffered a stroke and presented cognitive impairment due to other neurological diseases. In the case of closed homes, after three attempts, another subject was drafted from the list.

The first questionnaire answered was about demographic characteristics (gender, age, marital status and skin color), socioeconomic factors (education and income), labor (sitting work, or standing, crouching, lying, kneeling, vibration and/or trepidation, lifting weight, repetitive motion), lifestyle (physical activity, smoking, hours on TV, frequency on TV, hours at the computer, frequency on the computer) and type of diseases.

The position at work and type of movements (sitting, standing, crouching, lying, kneeling, vibration and/or trepidation, loading weight, repetitive movements) were characterized by the perception of the respondent identifying four options (never, rarely, usually, always), which best characterized the frequency of exposure.

The level of physical activity of the elderly was assessed using the International Physical Activity Questionnaire (IPAQ), short version, proposed by the World Health Organization (WHO) and Centers for Disease Control and Prevention (CDC). This instrument evaluates physical activities in leisure time, as moving from one place to another, household services and occupational activities.^7^

Pain in the lumbar spine was observed through the Nordic questionnaire, adapted to the Brazilian culture.^8^ Low back pain was defined as pain or discomfort in the last twelve months, not related to trauma or other problem. At the time of the interview the following question was asked to the elderly: "Have you had pain in the lumbar spine (lower back) in the last year?". For more specificity about the location of pain in addition to verbal questioning, an image of the regions of the vertebral column in different colors was presented, to identify the lumbar region. This type of tool is valid and reliable for measuring pain in the population, as it makes the individual very specific on locating the pain.^8^

The dependent variable was the functional capacity compromised due to musculoskeletal pain, measured by the Brazilian version of the Rolland-Morris questionnaire(Brazil) validated by Nusbaum.^9^ This instrument was translated into Portuguese language and adapted to Brazilian culture, presenting a high test-retest reliability (ICC 0.94) and inter-rater (ICC 0.95).^9^

Rolland-Morris contains a list of 24 phrases that the respondent indicates if the question describes his status on the day of application. The list, according to the author, has some phrases that people use to describe the pains they feel in the back. For each marked sentence one point is counted, so the total score ranges from zero to 24 points. The higher the total score, the greater the functional impairment of the individual. The minimum score is zero and represents no impact of pain on the person. There were defined as having low functional capacity individuals with scores greater than or equal to 14 in the Rolland-Morris questionnaire (cutoff that characterizes the presence of significant disability due to low back pain).^9^

### Data Analysis

The data were entered into a database and analyzes were stratified by gender using the statistical program SPSS, version 10.0 (SPSS, Chicago, USA). The analysis was performed using a descriptive and an analytical approach. In the descriptive approach distributions of absolute and relative frequencies for categorical variables were made and the analytical bivariate analysis was performed using the chi-square, and then, multivariate binary logistic regression, following the hierarchical model. The adopted method for the introduction of the variables in the models was the "backward stepwise". A significance level of 5% and a confidence interval (CI) of 95% were considered, with adjusted calculation of "odds ratios".^10^

## RESULTS

In Table 1, we observed that there was a higher frequency of elderly, of both genders, aged between 60 and 69 years (56.2% for men and 53.8% for women); 55.6% of men and 46.2% of women had 0-4 years of schooling; 50.3% of men and 76.3% of women were married; 66.6% of men and 77.4% of women had an income of 2-5 minimum wages; 51.6% of men and 50.5% of women reported having three or more diseases and 86.9% of men and 64.5% of women were sedentary. Of the elderly respondents, 70.9% were retired, and of these, 14.1% were still working by performing the same activities.

**Table 1:** Absolute and relative frequency of the sociodemographic variables, related diseases and level of physical activity of the elderly (Bauru, SP, 2011).

Factors	Gender	
	Masculine	Feminine
**Age group**		
60 - 69 years old	86 (56.2%)	50 (53.8%)
70 - 80 years old	67 (43.8%)	43 (46.2%)
**Years of schooling**		
0 - 4 years	85 (55.6%)	43 (46.2%)
5 - 8 years	68 (44.4%)	50 (53.8%)
**Ethnic group**		
White	77 (50.3%)	38 (40.9%)
Black	22 (14.4%)	12 (12.9%)
mulatto	54 (35.3%)	43 (46.2%)
**Marital status**		
Married	77 (50.3%)	71 (76.3%)
Widow, single, divorced	76 (49.7%)	22 (23.7%)
**Income**		
Up to 1 minimum wage	52 (34.0%)	21 (22.6%)
Two to five minimum wages	101 (66.6%)	72 (77.4%)
**Related diseases**		
Up to two	74 (48.4%)	46 (49.5%)
Three or more	79 (51.6%)	47 (50.5%)
**Level of physical activity**		
Sedentary	133 (86.9%)	60 (64.5%)
Active	20 (13.1%)	33 (35.5%)

Regarding the variables related to work (Table 2) there have been higher percentages in work activities that individuals performed in the standing position (95.4% for men and 92.5% for women), standing leaning forward (93.5% for men and 89.2% for women), repetitive movements (77.1% for men and 98.2% for women) and lifted and carried weight (62.1% men and 81.7% women).

**Table 2:** Absolute and relative frequency according to variables related to work of the elderly (Bauru, SP, 2011).

Factors	Gender	
	Masculine	Feminine
**Repetitive movements**		
Always/usually	118 (77.1%)	83 (98.2%)
Never/rarely	35 (22.9%)	10 (10.8%)
**Vibration/trepidation**		
Always/usually	44 (28.8%)	55 (59.1%)
Never/rarely	109 (71.2%)	38 (40.9%)
**Lifting and caring weight**		
Always/usually	95 (62.1%)	76 (81.7%)
Never/rarely	58 (37.9%)	17 (18.3%)
**Kneeling position**		
Always/usually	87 (56.9%)	47 (50.5%)
Never/rarely	66 (43.1%)	46 (49.5%)
**Siting position**		
Always/usually	41 (26.8%)	28 (30.1%)
Never/rarely	112 (73.2%)	65 (69.9%)
**Siting lifting weight**		
Always/usually	19 (12.4%)	17 (18.3%)
Never/rarely	134 (87.6%)	76 (81.7%)
**Siting and leaning body**		
Always/usually	50 (32.7%)	29 (31.2%)
Never/rarely	103 (67.3%)	64 (68.8%)
**Standing position**		
Always/usually	146 (95.4%)	86 (92.5%)
Never/rarely	7 (4.6%)	7 (7.5%)
**Standing position leaning forward**		
Always/usually	143 (93.5%)	83 (89.2%)
Never/rarely	10 (6.5%)	10 (10.8%)

The mean score on the assessment of functional capacity, obtained in the Roland-Morris disability questionnaire was 10.46 ± 5.62. A fraction of 67.5% of the elderly had no adequate functional capacity while 32.5% had. On the responses to the Roland-Morris, the most frequently mentioned items were:

"I change my position" with 98 annotations for men and 57 for women; "I walk slower" with 110 and 62 annotations, respectively; "I use handrails to climb stairs" with 97 and 60 annotations; " I need support to rise from a chair" with 83 and 46;

"I dress up more slowly" with 101 and 67; "I stand for short periods" with 107 and 41; "I try not to bend or crouch" with 99 and 68; "I feel pain most of the time" 90 and 53; "I face problems to put on socks" with 84 and 49; "I avoid heavy work at home" with 125 and 76, and "I climb stairs more slowly" with 100 and 70 annotations, respectively.

Table 3 shows significant associations between inadequate functional capacity and age, household income, reported illnesses and physical activity level, indicated by the *x* 2 test.

**Table 3:** Bivariate analysis of the functional capacity in relation to sociodemographic variables, diseases and level of physical activities of the elderly (Bauru, SP, 2011).

Factors	Functional capacity		x^2^ test
	Inadequate	Adequate	
**Gender**			
Masculine	100 (60.2%)	53 (66.2%)	*p > 0.05*
Feminine	66 (39.8%)	27 (33.8%)	
**Age group**			
60 - 69 years old	81 (48.8%)	51 (63.8%)	*p < 0.05*
70 - 80 years old	85 (51.2%)	29 (36.2%)	
**Years of schooling**			
0 - 4 years	45 (56.2%)	83 (50.0%)	*p > 0.05*
5 - 8 years	35 (43.8%)	83 (50.0%)	
**Ethnic group**			
White	36 (45.0%)	79 (47.6%)	*p > 0.05*
Black	11 (13.8%)	23 (13.9%)	
mulatto	33 (41.3%)	58 (38.5%)	
**Marital status**			
Married	46 (57.5%)	101 (60.8%)	*p > 0.05*
Widow, single, divorced	34 (42.5%)	65 (39.2%)	
**Income**			
Up to 1 minimum wage	30 (37.5%)	43 (25.9%)	*p < 0.05*
Two to five minimum wages	50 (62.5%)	123 (74.1%)	
**Related diseases**			
Up to two	93 (56.0%)	27 (33.8%)	*p < 0.05*
Three or more	73 (44.0%)	53 (66.2%)	
**Level of physical activity**			
Sedentary	70 (87.5%)	138 (83.1%)	*p < 0.05*
Active	10 (12.5%)	28 (16.9%)	
Active			

Also using the *x* 2 test, it was possible to identify, in Table 4, significant associations between inadequate functional capacity and repeated movements and the sitting position.

**Table 4:** Bivariate analysis of the functional capacity in relation to work characteristics (Bauru, SP, 2011).

Factors	Functional capacity		x^2^ test
	Inadequate	Adequate	
**Repetitive movements**			
Always/usually	131 (78.9%)	70 (87.5%)	*p < 0.05*
Never/rarely	35 (21.1%)	10 (12.5%)	
**Vibration/trepidation**			
Always/usually	94 (56.6%)	53 (66.2%)	*p > 0.05*
Never/rarely	72 (43.4%)	27 (33.8%)	
**Lifting and caring weight**			
Always/usually	47 (28.3%)	28 (35.0%)	*p > 0.05*
Never/rarely	119 (71.7%)	52 (65.0%)	
**Kneeling position**			
Always/usually	79 (47.6%)	33 (41.2%)	*p > 0.05*
Never/rarely	87 (52.4%)	47 (58.8%)	
**Siting position**			
Always/usually	125 (75.3%)	52 (65.0%)	*p < 0.05*
Never/rarely	41 (24.7%)	28 (35.0%)	
**Siting lifting weight**			
Always/usually	142 (85.5%)	68 (85.0%)	*p > 0.05*
Never/rarely	24 (14.5%)	12 (15.0%)	
**Siting and leaning body**			
Always/usually	117 (70.5%)	50 (62.5%)	*p > 0.05*
Never/rarely	49 (29.5%)	30 (37.5%)	
**Standing position**			
Always/usually	10 (06.0%)	4 (05.0%)	*p > 0.05*
Never/rarely	156 (94.0%)	76 (95.0%)	
**Standing position leaning forward**			
Always/usually	14 (08.4%)	6 (07.5%)	*p > 0.05*
Never/rarely	152 (91.6%)	74 (92.5%)	

The results of logistic regression analysis (Table 5) showed that age (*p* = 0.001), the amount of reported diseases (*p* = 0.02) and the level of physical activity (*p* = 0.04) showed independent association with inadequate functional capacity.

**Table 5:** Multivariate analysis of logistic regression. Final model for independent associations with inadequate functional capacity (Bauru, SP, 2011).

Factors	p value	Adjusted OR*/CI 95%
**Age group**		
60 - 69 years old	0.001	1.00
70 - 80 years old		2.25 (1.19 – 4.21)
**Related diseases**		
Up to two	0.02	1.00
Three or more		1.39 (1.22 – 1.72)
**Level of physical activity**		
Sedentary	0.04	1.00
Active		2.71 (1.34 – 5.47)

## DISCUSSION

It has been observed in this study a higher frequency of elderly aged between 60 and 69 years old, married, with low education and income of two to five minimum wages, which concurs with other research conducted with the elderly.^11^^,^ 12

Regarding work, it was noticed that the elderly, in their occupational activities, performed "always/usually" repetitive movements, lifting and carrying weight and worked in a standing position and standing leaning forward. In Pelotas it was observed that most of the population of this age group worked in a sitting position, with repetitive movements, vibration and/or trepidation and carrying weight.^13^

In the present study, 67.5% of the elderly had no adequate functional capacity averaging 10.46 ± 5.62 points on the Roland-Morris disability questionnaire, like other research on the same topic.^14^^-^ 16

Also in this study it was found that the elderly aged 70 or older were 2.25 times more likely to have not adequate functional capacity. Other authors have noticed increased disability in older ages.^17^^,^ 18 With advancing age the human body begins a slow degenerative process, resulting in the gradual decrease in functional capacity that may lead the elderly to disability, with loss of cognitive domain and physical dysfunction which contribute to the reduction of his autonomy.^19^

Regarding the level of physical activity, sedentary elderly had 2.21 times the odds of not having adequate functional capacity. In Canada it was noticed that sedentary subjects had 33% higher odds of functional disability than active ones,^20^ and in the United States association was found between low functional capacity and low level of physical ability among participants of a survey with individuals suffering from low back pain.^21^ Physical activity is an important predictor of functional capacity in patients with low back pain. The more active elderly lives better, because physical activities allow the preservation and minimization of debilitating bodily pains and organic changes. It is important to consider that, although the physical performance is modified over the years, with proper and regular practice of physical activity, respecting the biological individuality, these modifications will be restricted, favoring the extension of life, enriching the quality of life, contributing to rehabilitation of interdependent physiological functions.^22^

Seniors with three or more diseases showed 39% more chances of inadequate functional capacity. These data were confirmed in the city of Guatambu, SC, Brazil, where the elderly who were affected by five or more comorbidities had a prevalence of 2.84 times less likely to have an adequate functional capacity.^23^ A population-based study in São Paulo, Brazil,^24^ showed that chronic diseases have a strong influence on the functional capacity of the elderly. The presence of hypertension increases by 39% the chance of the elderly being dependent in instrumental activities of daily living (IADL), heart disease increases by 82%, arthropathy in 59% and pulmonary disease in 50%. For the IADL dependency and activities of daily living (ADL), the odds more than doubled for the presence of each of these chronic diseases. Chronic non-communicable diseases are more common among the elderly, and are the most disabling. Successful aging becomes the result of the interaction of multi-dimensional factors, which include items related to physical health, mental issues, independence in daily life, economic and psychosocial factors.^21^

One limitation of this study refers to the cross-sectional design that did not allow establishing a temporal relationship of cause and effect between variables. Another point is the sample design that investigated the prevalence of inadequate functional capacity in only one region of low social class, where there are high rates of individuals with low educational level, hindering a more heterogeneous sample, ideal for this type of analysis.

## CONCLUSION

In the population investigated in this study, we observed a significant association between inadequate functional capacity in the elderly over 70 years old, with reports of three or more diseases and sedentary. These data may be useful as preventive strategies in health units and encourage changes in routine care to the elderly health, investing in preventive actions.
